# Knowledge and attitude of key community members towards tuberculosis: mixed method study from BRAC TB control areas in Bangladesh

**DOI:** 10.1186/s12889-015-1390-5

**Published:** 2015-01-31

**Authors:** Sukanta Paul, Rahima Akter, Afzal Aftab, Antora M Khan, Mrittika Barua, Shayla Islam, Akramul Islam, Ashaque Husain, Malabika Sarker

**Affiliations:** James P Grant School of Public Health, BRAC Institute of Global Health, BRAC University, 68 Mohakhali, Dhaka, 1212 Bangladesh; Health Nutrition and Population Programme, BRAC, 75 Mohakhali, Dhaka, 1212 Bangladesh; National Tuberculosis Control Program, Directorate General of Health Services, Mohakhali, Dhaka, 1212 Bangladesh

**Keywords:** Tuberculosis, Key community member, Key informant, Knowledge, Attitude, Advocacy, Communication, and Social Mobilization (ACSM) activities, Bangladesh

## Abstract

**Background:**

Bangladesh National Tuberculosis (TB) Control Programme adopted a number of strategies to facilitate TB diagnosis and treatment. ‘Advocacy, Communication and Social Mobilization’ (ACSM) was one of the key strategies implemented by BRAC (Bangladesh Rural Advancement Committee, a non-governmental development organization) TB control program. The purpose of this study is to assess the knowledge and attitudes of the key community members (KCMs) participated in ACSM in BRAC TB control areas.

**Methods:**

This study combined quantitative and qualitative methods using a mixed method approach. KCMs in three districts with low TB case detection rates were targeted to assess the ACSM program. The quantitative survey using a multi-stage random-sampling strategy was conducted among 432 participants. The qualitative study included in-depth interviews (IDIs) of a sub sample of 48 respondents. For quantitative analysis, descriptive statistics were reported using frequencies, percentages, and Chi square tests, while thematic analysis was used for qualitative part.

**Results:**

Most (99%) of the participants had heard about TB, and almost all knew that TB is a contagious yet curable disease. More than half (53%) of the KCMs had good knowledge regarding TB, but BRAC workers were found to be more knowledgeable compared to other KCMs. However, considerable knowledge gaps were observed among BRAC community health workers. Qualitative results revealed that the majority of the KCMs were aware about the signs, symptoms and transmission pathways of TB and believed that smoking and addiction were the prime causes of transmission of TB. The knowledge about child TB was poor even among BRAC health workers. Stigma associated with TB was not uncommon. Almost all respondents expressed that young girls diagnosed with TB.

**Conclusions:**

This study finding has revealed varying levels of knowledge and mixed attitudes about TB among the KCMs. It also provides insight on the poor knowledge regarding child TB and indicate that despite the significant success of the TB program stigma is yet prevalent in the community. Future ACSM activities should engage community members against stigma and promote child TB related information for further improvement of BRAC TB Control Programme.

**Electronic supplementary material:**

The online version of this article (doi:10.1186/s12889-015-1390-5) contains supplementary material, which is available to authorized users.

## Background

Tuberculosis (TB) remains a major public health issue in many developing nations. The current global estimate is that over 8.8 million TB cases emerge each year, and nearly 1.5 million people die from TB yearly: 98% of these cases and deaths occur in developing countries [1]. Poor public awareness, coupled with a limited engagement of communities and non-governmental organizations, has been identified as a challenge that impedes TB control [2]. Geographically, the burden of TB is highest in Asia and Africa [3]. Bangladesh is among the top-10 high TB burden countries for all forms of TB. The prevalence is 411/100 000 and the incidence is 225/100 000 population per year [1]. To reduce this burden, the National TB Control Programme (NTP) has adopted the Directly Observed Treatment, Short-course (DOTS) strategy, delivered primarily through government-run health facilities [4]. However, major obstacles to implementation remain, primarily owing to insufficient infrastructure and a shortage of appropriately trained health personnel [5,6]. To address these challenges, the World Health Organization recommends the utilization of an Advocacy, Communication and Social Mobilization (ACSM) framework for national TB Control Programmes. This strategy framework addresses four key challenges: improving case detection and treatment adherence, reducing stigma and discrimination, empowering TB patients, and mobilizing the resources and political commitment required to combat TB [7].

As part of ACSM, in 2004 BRAC (Bangladesh Rural Advancement Committee, a non-governmental development organization) TB Control Programme started a nationwide mass campaign program. A component of the programme was an orientation on TB for different stakeholders, including cured TB patients, community leaders (headmaster, Union Parishad (UP) member, businessmen, religious leaders, and factory workers/owners), different women’s group, girl guides and scouts, private medical practitioners, intern doctors, village doctors (VDs), drug sellers (DSs) and health workers [8]. Community leaders such as these play a crucial role in the community because their decisions and opinions are well respected and honoured [9]. The community-based social mobilization effort commenced in order to expand knowledge about the diagnosis, treatment, and prevention of TB and to increase awareness about the widespread availability of directly observed treatment for Tuberculosis [10].

Until now, the BRAC TB Control Programme has not conducted an assessment on ACSM. It is important to appraise TB related knowledge and attitudes of the community members to help BRAC modify the ACSM strategy to make it more effective. This paper presents insight from the study of TB related knowledge and attitudes of different key community members (KCMs) in rural Bangladesh.

## Methods

### Study design and setting

This study combined quantitative and qualitative methods, leading to a mixed method approach [11]. The study was conducted in two phases. The first phase included a quantitative survey and the second phase a qualitative study consisting of in-depth interviews (IDIs). The three districts with low TB case detection rate compared to other districts based on the MIS report (unpublished) BRAC TB control program were first selected and then twelve unions of 6 *upazilas* (sub districts) of those districts were selected as study sites. The case detection rate was 85% on average in these three districts compared to 110% in other areas (Unpublished data from TB case finding report, BRAC MIS 2012). The expected target for case detection is set by the BRAC TB Control Programme.

### Study population

The study participants were selected from the KCMs listed for ACSM of BRAC TB control programme. These lists were collected from regional BRAC offices. The types of KCMs targeted are the following: BRAC Shasthya Shebikas (SS), BRAC Health staffs (Shasthya Kormi-SK), program organizers (POs), lab programme organizers (lab PO), drug sellers (pharmacy-based), village doctor (practicing allopathic medicine without a formal degree), DOTS providers (local people who provide DOTS on request), *Imams* (Islamic leaders), *Muezzins* (appointed mosque authorities who lead and recite the call to prayer for every event of prayer and worship), headmasters of primary and secondary schools, Union Parishad Members (elected local government), cured TB patients-CTP (TB patient who have completed treatment and are cured), businessmen (locally respected), social workers (people who are involved with social welfare), factory workers/owners, performers of folk songs and members of popular theatre groups.

### Study period

The study was carried out from May 2013 to July 2013.

### Sampling frame

A multi-stage random sampling design was used to select study areas. Stage one involved randomly selecting three districts from areas with a low TB case detection rate, as assessed by the BRAC TB control programme. The second stage consisted of randomly selecting two sub-districts from each district. In the third stage, two unions were selected randomly from each sub-district. All random draws were made using a random number table. In final stage KCMs targeted for ACSM of BRAC TB program from 12 unions were selected as study subjects. However, the study subjects were chosen purposively because number and category of KCMs were dependent on the presence of certain institution (such as school, mosque) or professions (folk singer, social worker) in that specific union. As we were limited to the actual KCMs who live in selected areas, we missed recruiting different categories of KCMs due to the random selection of unions. The criteria for purposive selection were profession gender and level of knowledge.

A sample size of 432 was required, which was calculated with the prevalence of good knowledge (able to respond correctly 10 questions) is 50%, 80% power, error rate 5%, and design effect 1.04. In total 432 KCMs, 36 from each union and144 from each district were recruited.

After completion of the quantitative survey, a qualitative study was carried out in the same districts among 48 respondents (16 respondents from each district). Respondents were selected purposely based on their profession and responses to knowledge question in the quantitative survey. The qualitative data were collected through guided IDIs (See Additional file 1). The content of the guideline included signs and symptoms, transmission, prevention and treatment of TB. IDIs were conducted with 12 SSs, twelve CTPs, six *imams*, 6 headmasters, six VDs and 6 DSs.

### Data collection

The quantitative survey questionnaire was administered by 36 enumerators under the guidance of 6 research assistants supervised by the first and second author. Prior to data collection, a one day extensive training session was conducted which included a background briefing on the project and its objective. The training also disseminated information on ACSM activities undertaken by the BRAC TB control programme. The questionnaire was prepared in English (See Additional file 2) and translated into Bengali (local language). After pre-testing, the Bengali version of the questionnaire was modified and back translated into English, incorporating the feedback from the pre testing. All of the instructions, including skipping and probing were documented as a field protocol to assist the field workers.

Six qualitative researchers along with two other authors were involved in qualitative data collection. Responses to the IDIs were made both through notes during interview and audio recordings.

### Quantitative analysis

The questionnaire captured information on the socio demographics of the respondents. Knowledge about TB was assessed using 10 questions (See Additional file 3). Responses were categorized as “Good Knowledge” (10 correct answers), “Average Knowledge” (7–9 correct answers) and “Poor Knowledge” (less than 6 correct answers).

Data was entered into IBM SPSS Statistics software, version 20. After entry, data were checked carefully and cleaned in order to ensure quality.

For analysis, respondents were clustered into different categories: BRAC workers (SS, SK, PO and Lab-PO), informal health care providers (DS, VD, and DOTS providers), community leaders (*Imam*, *Muezzin*, Headmaster, UP Member), and community members (CTPs, businessmen, social workers, factory workers, performers of folk song and a popular theatre group). Descriptive statistics were reported using frequency and percentage and chi square tests was performed.

### Qualitative analysis

The qualitative data from the field were transcribed and translated from the digital recording. Data were analyzed independently by the research team to ascertain inter-rater coding reliability [12]. Following the initial review of the data, a categorising system was developed through using thematic analysis based on the study objectives and interview guidelines. Then the authors identified themes and sub-themes emerging from the data analysis and finally produced a final thematic index to code all the data.

### Ethical approval

Approval for the study was obtained through the Ethical Review Committee of James P. Grant School of Public Health. Verbal consent was obtained from each respondent after clarifying the confidentiality and voluntary participation features of the study.

Furthermore, the study questionnaire and guideline were anonymous and interviews were conducted in a private setting to preserve privacy of the respondents for sensitive questions.

## Results

### Quantitative

#### Socio demographic profile

A total of 432 participants with response rate of 100% were interviewed for this study, 181 (42%) were males and 251 (58%) were females, 259 (60%) were ≤40 years old, and 147 (34%) had a monthly income of BDT (Bangladeshi Taka) 4001–8000 (51–103 USD) (Table 1).

**Table 1:** **Knowledge level of the key community members by demographic profile (N = 432)**

	**Knowledge level of the key community members**			***χ*** ^***2***^
	**Good**	**Average**	**Poor**	
	**n (%)**	**n (%)**	**n (%)**	
Age				
≤40	145 (33)	108 (25)	5 (1)	0.134
>40	85 (20)	81 (19)	8 (2)	
Sex				
Male	80 (19)	91 (21)	10 (2)	0.001*
Female	150 (35)	98 (23)	3 (1)	
Educational status				
Never attended	23 (5)	26 (6)	-	0.175
≤10 years of education	140 (32)	124 (29)	9 (2)	
>10 years of education	67 (16)	39 (9)	4 (1)	
Marital status				
Married	192 (44)	155 (36)	10 (2)	0.69
Unmarried	21 (5)	14 (3)	1 (1)	
Single	17 (4)	20 (5)	2 (1)	
Monthly income				
<4000	59 (14)	50 (12)	3 (1)	0.45
4001-8000	72 (17)	72 (17)	3 (1)	
8001-12000	50 (12)	38 (9)	5 (1)	
12001-16000	23 (5)	11 (3)	-	
>16001	26 (6)	18 (4)	2 (1)	

*p < 0.001.

### Knowledge level

Overall, 229 (53%) of all KCMs had good knowledge regarding TB, and only 13 (3%) of them had poor knowledge. BRAC workers were more knowledgeable compared to other groups of KCMs (Table 2).

**Table 2:** **Knowledge level of the key community members by category (N = 432)**

**Category of the key community members**	**Subcategory of the key community members**	**Knowledge level of the key community members**		
		**Good**	**Average**	**Poor**
		**n (%)**		
BRAC worker	Shasthya Shebika	105 (59)	72 (40)	2 (1)
	Shasthya Kormi	14 (70)	6 (30)	-
	Program Organizer	15 (88)	2 (12)	-
	Lab Program Organizer	8 (89)	1 (11)	-
Informal health care provider	Drug Seller	17 (52)	12 (36)	4 (12)
	Village Doctor	19 (58)	13 (39)	1 (3)
	DOTS Provider	5 (71)	2 (29)	-
Community leader	Imam	7 (28)	15 (60)	3 (12)
	Muezzin	-	1 (100)	-
	Head Master	11 (55)	9 (45)	-
	UP Member	9 (32)	19 (68)	-
Community members	Cured TB Patient	13 (35)	24 (65)	-
	Businessman	3 (42)	2 (29)	2 (29)
	Social worker	1 (33)	2 (67)	-
	Factory Worker	-	2 (67)	1 (33)
	Folk song and popular theatre group	3 (30)	7 (70)	-

Ninety-nine percent of the respondents had heard about TB and knew that TB is a contagious disease but curable and early treatment is crucial. However, 78 (18%) respondents were unaware of the symptoms of TB and 17 (4%) believed that TB could not be transmitted during treatment. In addition, 73 (17%) respondents did not know that a child can also be infected with TB (Data not shown).

The only explanatory variable significantly associated with the knowledge level of TB was sex of respondent, 151 (35%) female respondents had “Good knowledge” about TB (Table 1), compared to 80 (19%) of male respondents.

With regard to the source of knowledge, 358 (21%) reported BRAC staff as their major source of knowledge. Otherwise, mass media [340 of them stated television (20%), 182 of them mentioned radio (11%), 151 of them reported poster (9%), and 142 of them stated the newspaper (8%)] was reported as a source of information (Table 3).

**Table 3:** **Key community members source of knowledge on TB**

**Source of knowledge**	**n (%)**
BRAC staff	358 (21)
Television	340 (20)
Radio	182 (11)
Poster	151 (9)
Newspaper	142 (8)
Private clinic	144 (8)
Community people	140 (8)
Government health staff	82 (5)
Others	60 (4)
Cured TB patient	57 (3)
Other non-governmental organizations	46 (3)

The present study revealed several BRAC Shasthya Shebikas (BRAC community health volunteerts) have a knowledge gap. Of the total SS (179), 19 (11%) could not mention the signs and symptoms of TB, did not know that a person can suffer from TB more than once in a lifetime, and that children can also be infected with TB. Moreover, 30 (17%) SS did not know that one should continue treatment when symptoms persist for more than three weeks. Fourteen (8%) of them did not know that six months of treatment is essential for cure and eight (4%) did not know that TB can be transmitted during treatment.

### Qualitative

#### Knowledge on TB

The majority of the KCMs knew the signs and symptoms of TB. Most notably, SS and informal health care providers mentioned that coughing for more than three weeks with phlegm and blood is a major sign and symptom of TB. They also mentioned fever in general and at night, loss of appetite, weight loss, night sweating, and chest pain to be additional main signs and symptoms of TB. Few mentioned coughing up blood and some pointed out fever as a primary sign and symptom of TB.

“...... *I have been working as a SS for the last 5 years. I know the symptoms (Lokkhon) of TB very well. If I didn’t know then how could I have worked for so long at BRAC. If I know, then only people will know. There are many symptoms of TB, such as a persistent cough for more than three weeks, fever, night sweating, lack of appetite and weight loss. Beside these there can be more symptoms” (Shasthya Shebika, Age 45).*

Differences have been found between the various categories of the KCMs in response to knowledge on how TB is transmitted. Some respondents believed TB spreads via air droplets and sharing utensils with another TB patient. Most of them mentioned air droplets while sneezing or coughing and living in close proximity as the source of TB transmission. The majority of the KCMs considered smoking or addiction was one of the prime causes for getting infected with TB. However, all SS affirmed the fact that both males and females have an equal chance of becoming infected with TB. However, many KCMs felt that men are the main at-risk population of acquiring TB. As stated by one of the female respondents:

*“Benapole is a border area between Bangladesh and India. We live in such a place where all kinds of drugs are easily obtainable. Illegal operations are frequent over here. Young adolescents in this area take drugs because it is affordable and easily accessible. In addition, most of them smoke cigarettes as well. So they are more vulnerable to be infected by TB” (Cured TB Patient, Age 35).*

The majority of the respondents knew that TB can be transmitted during treatment and some of them cited malnutrition, unhealthy environment and unawareness as risk factors for TB. Except for a few *imams,* all KCMs believed that TB is curable. Moreover, many of the KCMs told that TB is curable if a 6 month treatment regimen is completed. The majority of respondents asserted that TB can happen more than once in a lifetime, even after successful completion of treatment, but early treatment is required if someone becomes affected with TB.

The respondents reported that the key source of information was BRAC SSs and at this point the majority of people in Bangladesh know about TB. As noted by one of the respondents:

*“…..Yes, I have heard about TB. It’s a fatal disease and it can finish life. I don’t know a lot regarding this. A lot of people died before because of TB. But I have heard a lot of things from the young girls used to move one household to another wearing white apron. They often come to my house and spoke to my wife. Besides this once I participated in a meeting of BRAC last year. They have discussed about TB in the meeting. I can hardly recall what they discussed there about TB. But I think TB and Jokkha (Bengali term of TB) is quite different in terms of sign and symptoms. Extreme fever is the major symptom of Jokkha whereas TB has a lot of symptoms. BRAC gives the treatment for TB” (Imam, Age 50).*

Only a few KCMs knew about childhood TB. Among them a few SSs told that they had prior knowledge about childhood TB. But only three of them had ever identified child TB patient.

### Attitude and beliefs

Respondents reported feeling humiliated when any family member became infected with TB. KCMs reported that community members did not want to disclose if any family member had TB. While asking about the gender differences almost all respondents presented that it is difficult for girls to get married if she is identified to have TB. Several SS mentioned that husbands tend to leave their wives, if wives are diagnosed with TB. The majority of the SSs suggested that family members of a TB patient do not interact with the patient and keep distance from him/her while using utensils, clothing and doing other household related activities because of the fear of transmission. Moreover, CTPs also mentioned the same. The majority of imams and headmasters mentioned that people are often excluded if they have TB. This scenario was reported to be harsher for women.

*“There was a girl in our community whose father had TB. When her proposal came for marriage, someone from the neighborhood informed the groom’s family that bride’s father was a TB patient. As a result of that the marriage was cancelled by the grooms family” (Shasthya Shebika, Age 45).*

“…..*It is difficult to marry off a girl with TB. So people tend to hide it. Such a problem is not that serious for men, but a woman will not marry a man if it is known that her would-be husband has TB. On-going marriages can also be broken” (Village doctor, Age 28).*

However DSs and VDs had a different view, they rarely witnessed gender discriminations among TB patients. Several mentioned there might be misconceptions about TB amongst the people living in the remote places of the villages who are ignorant about the disease. Another KCM expressed satisfaction with the changing attitude of people towards TB.

*“Yes, I have seen such incident happen in Chougacha (an union). A marriage proposal came for a girl who was later known to be a TB patient. The groom’s side at first refused the proposal when they heard about it. But they agreed later though and the girl got married after she was cured… beside these, disease is given by Allah (God), why should we look at someone differently” (Village doctor, Age 33).*

## Discussion

This paper offers insight on the current knowledge regarding TB among the KCMs who participated in ACSM program in three BRAC TB control areas in Bangladesh. A large proportion of the KCMs had good knowledge on TB but several had poor knowledge. Our findings suggest that informal healthcare provider and BRAC community health workers are more knowledgeable in comparison to other KCMs. Another nationwide study in Bangladesh reported that 49% of untrained informal allopathic providers (Village Doctors and Drug Shop Attendants) have better knowledge of TB [13]. In terms of knowledge regarding transmission, the majority of the interviewed subjects mentioned that TB can be transmitted through air droplets. These views are in line with a study from Pakistan and Egypt, where most of the respondents expressed the view that TB spreads through the air when coughing or sneezing [14,15]. In line with this, the findings from the qualitative component revealed that the majority of the KCMs of this study identified a cough for more than three weeks to be the major indicator of TB, which is similar to a study in Pakistan [16]. It is important, particularly for KCMs to know this major symptom and mode of transmission of TB as they can influence behavior like cough etiquette and respiratory hygiene, which are critical in preventing TB transmission [17]. The correct information on signs and symptoms is vital for early diagnosis and subsequent enrollment for treatment, and is the most effective for TB control.

The findings from this study also confirmed that health providers like village doctors have better knowledge of sign-symptoms, transmission and prevention of TB that is consistent with another recent study conducted in 30 sub-districts in rural Bangladesh [13]. Health providers’ knowledge is an important facilitating factor for improved case detection and patients’ timely referral. It is proven that health care providers’ knowledge levels directly impact the effectiveness of healthcare services [18,19].

Additionally, knowledge on the association of TB with smoking was commonly mentioned by the KCMs. The majority of the KCMs mentioned smoking has a strong association with transmission of TB, which is consistent with other studies which reported that smoking and drinking alcohol are additionally perceived causes of TB and those who smoke cigarettes do in fact have nearly twice the risk of TB than non-smokers [8,20,21]. Up to one in five deaths from TB could be avoided if patients were not smokers [22].

One of the main sources of knowledge on TB listed by respondents was health provider, particularly BRAC health worker, which is consistent with the findings of a study conducted in Pakistan [16] and south western Ethiopia [23]. This indicates that BRAC has been successful in reaching out to communities. Yet this study identified a significant knowledge gap regarding the common modalities of TB among BRAC community health workers ‘SS’, which is also similar to a study conducted in Peru [24]. This in turn might negatively affect the health care seeking behavior of the community and delay diagnosis and treatment.

Mass media was found to be another key source of information, consistent with studies done previously in Bangladesh [25], Lahore [26], and Pakistan [14]. BRAC and Government of Bangladesh invest significantly for mass awareness through the media and one of the important messages it conveys is ‘TB is a curable disease’. This may also be the reason that almost all of the KCMs could mention that TB can be cured completely.

This study found KCMs’ knowledge about TB to be significantly associated with gender, “good knowledge” of TB was observed more in the female participants. It is important to note here that most of the female participants in the current study were SSs and the SSs are BRAC workers and trained in TB. Nevertheless, good knowledge of the nature of the disease among the frontline health workers may also influence the attitude of the community towards TB and TB patients.

‘Childhood TB is emerging from the shadows’ [27], and an intriguing finding in this study was the poor knowledge about childhood TB among the KIs, in particular among SS, BRAC community health workers. One of the major reasons for poor detection of child TB in Bangladesh is poor knowledge of child TB among the health workers [28]. This has serious implication for child TB diagnosis. Globally, it has been increasingly recognized that TB cases among children (<15 years) are not well reported and recorded diagnoses fall short of actual prevalence rates [29]. According to the WHO in 2007, the proportion of child TB in low burden countries is 5% of all TB cases [8], in 2010, the Bangladesh National TB Program reported detection of 4236 child TB cases which is 2.7% of all diagnosed cases [30]. Thus, it is evident that in a high burden country like Bangladesh child TB cases are likely grossly under reported. A study from Bangladesh revealed that detection of childhood TB can be increased through awareness building [28]. Emphasis on child TB detection during training of health worker is therefore crucial.

The qualitative results revealed the presence of stigma associated with TB, which is severe for women. These views are in line with other studies conducted in India and Bangladesh [20,31,32] where most participants were of the view that TB can have adverse effects on the chances of getting married in females, but less so in males. Such stigmatization promotes social exclusion and distress for the infected person and their family.

One of the key strengths of this study is the utilization of a mixed method research method. The other strength is the diverse background of the study participants which is an indication of representation of the different sectors of that particular area. BRAC targeted all KCMs for ACSM activities and the study subjects were selected from the comprehensive list BRAC has provided. Nevertheless, several limitations are noteworthy. There is selection bias in the quantitative survey in that study subjects were not randomly recruited. If KCMs from high TB detection areas were included in this study, then we could have compared their level of knowledge with KCMs from low TB case detection areas, which would highlight whether ACSM activities played a major role in the success of the programme.

The TB control programme in Bangladesh cannot be deemed successful unless all the areas it covers display adequate performances. Based on the results of this study, the following recommendations from different stakeholders are significant and include: keep sensitizing the KCMs through ACSM activities, encourage the reduction of stigma against TB patients – with a focus on women, promote child TB related information, strengthen the training of health workers, and innovate in the use of media for mass awareness. The literature suggests that “community ‘participation’ enhances community ‘ownership’; in turn, ‘ownership’ leads to increased ‘capacity’ (or ‘competence’) and promotes program maintenance” [33,34]. All these components together can create sustainability within the national TB control programme.

## Conclusions

While literacy is the ultimate prevention to fight TB [35], there is still a need to educate the majority of key community members in Bangladesh about the etiology of TB and the appropriate preventive measures against it. The findings of this study, showing that KCMs display varied knowledge of and mixed attitudes towards TB, will be helpful for both the development and implementation of future TB ACSM activities in Bangladesh.

## Additional files

Additional file 1:
**In-depth interview guideline, doc, guideline used to assess the knowledge and attitudes of the key community members participated in ACSM program in three BRAC TB control areas in Bangladesh.**
Additional file 2:
**Baseline survey questionnaire, doc, tool used to assess the knowledge and attitudes of the key community members participated in ACSM program in three BRAC TB control areas in Bangladesh.**
Additional file 3:
**Reported responses of key community members regarding knowledge on TB, doc, descriptive report on frequency of all correct and incorrect responses for each of the 10 questions used for assessing knowledge about TB.**

## Abbreviations

ACSM: Advocacy Communication and Social mobilization
BRAC: Bangladesh Rural Advancement Committee
CTP: Cured Tuberculosis Patient
DOTS: Direct Observed Treatment, Short-course
DS: Drug Seller
HH: Household
IDI: In depth Interview
KCM: Key Community Member
LAB PO: Lab Program Organizer
NGO: Non-Governmental Organization
NTP: National Tuberculosis Programme
PO: Program Organizer
SK: Shasthya Kormi – (Female, salaried supervisor of the SS)
SS: Shasthya Shebika– (The term used in BRAC Bangladesh Health, Nutrition and Population Programme)
TB: Tuberculosis
VD: Village Doctor
WHO: World Health Organization
