# Supplementary material for: Knowledge and attitude of key community members towards tuberculosis: mixed method study from BRAC TB control areas in Bangladesh
Source: BMC Public Health. 2015 Jan 31;15:52. doi: 10.1186/s12889-015-1390-5 (PMC4322444; doi:10.1186/s12889-015-1390-5)
Supplement: Additional file 1: — In-depth interview guideline, doc, guideline used to assess the knowledge and attitudes of the key community members participated in ACSM program in three BRAC TB control areas in Bangladesh. [file 12889_2015_1390_MOESM1_ESM.docx]

participant INFORMED CONSENT FORM

In Depth Interview

**Knowledge and attitude of key community members in three BRAC TB control areas**

Greetings, I am _________________________ and this is *(If there are two interviewers)*. I am/we are working in a research project under James P. Grant School of Public Health at BRAC Institute of Global Health, BRAC University.

We are conducting a study on the key community members such as BRAC staffs, Religious leaders, Village Doctors, Drug Sellers, Headmasters and beneficiaries (Cured TB Patient) who have participated in the Advocacy, Communication and Social Mobilization (ACSM) activities of BRAC TB control Programme. In this study, we wish to assess your knowledge and attitude towards TB. We also want to know about your experience, perception and opinion about TB. The information you provide will be used to improve TB Control Programme in Bangladesh.

We invite you to participate in this study. You will have to answer some questions in the subject explained above. The interview will not take more than 40-45 minutes. Your responses will be kept confidential and will not be used for any other purpose other than this study. You can ask for any clarification of any question and can withdraw from the study anytime you want.

Do you have any further inquiry about this study? Yes No

(*If “Yes” please answer to any other inquiry of the respondent)*

Do you agree to participate in this study? Yes No

**In depth Interviews (Baseline Study)**

**May-July 2013**

| **Age:** | **Sex:** |
| --- | --- |
| **District Name:** | **Area/address:** |
| **Date of interview:** | **Interviewer name:** |
| **Time interview started:** | **Time interview ended:** |

**General Background**

1. How long have you lived in this community/village?
2. How many people do you have in your family?
3. Where do you usually go if you have general illness?

**Knowledge on TB**

1. What do you know about TB? Please explain.
2. Do you know the sign and symptoms of TB? ***Probe:*** Types of symptoms.
3. After how long if these symptoms persist one should seek treatment? ***Probe:*** Why?
4. How long a person should take treatment to get cured from TB?
5. Do you know how TB can be transmitted? Please explain.
6. How can a person become affected by TB? ***Probe:*** Who (Male/female/child) can be affected with TB according to you? Why?
7. Do you think that a person can have TB more than once in lifetime? ***Probe:*** Why?

**Attitudes and Stigma**

1. How would you know that you have TB?
2. What would be the first thing came to your mind if you heard that you have TB?
3. What would be your reactions if you were diagnosed with TB? ***Probe:*** worried/anxiety.
4. How does a person with TB being treated in your community? ***Probe:*** Positively/negatively, ask if there is any gender differences observed, request him/her to tell 1 or 2 story from their experience.

**Closing Questions**

1. Do you feel there is something important we should have asked that we did not address?

***[Thank you very much for your time]***
