# Supplementary material for: Knowledge and attitude of key community members towards tuberculosis: mixed method study from BRAC TB control areas in Bangladesh
Source: BMC Public Health. 2015 Jan 31;15:52. doi: 10.1186/s12889-015-1390-5 (PMC4322444; doi:10.1186/s12889-015-1390-5)
Supplement: Additional file 2: — Baseline survey questionnaire, doc, tool used to assess the knowledge and attitudes of the key community members participated in ACSM program in three BRAC TB control areas in Bangladesh. [file 12889_2015_1390_MOESM2_ESM.docx]

**Knowledge and attitude of key community members in three BRAC TB control areas**

**Baseline data collection**

**May – July 2013**

**Survey objective:** To assess the knowledge and attitudes of the key community members participated in ACSM program in three BRAC TB control areas in Bangladesh

**CONSENT**

*(Please read out to the respondent before starting interview)*

Assalamu Alaikum, my name is __________. I am working with James P Grant School of Public Health, BRAC Institute of Global Health, BRAC University, Dhaka.

We are conducting an assessment survey on BRAC Tuberculosis Control Programme. We wish to learn about your knowledge and attitudes regarding tuberculosis (TB). We hope to understand your needs and the best way to bring information to you, as well as barriers to seeking medical care. The information you provide will be used to improve BRAC TB control Programme.

Your answers will not be released to anyone and will remain anonymous. Your name will not be written on the questionnaire or be kept in any other records. Your participation is voluntary and you may choose to stop the interview at any time.

Do you agree with the interview? Yes = 1 No = 2

Do you have any questions on this? Yes = 1 No = 2

If yes, please specify ………………………………………………………………

Should I proceed? Yes = 1 No = 2

Thank you for your assistance.

***Interviewer:*** *Do not read the responses unless the direction indicate*

**ID: |______| - |________| - |___|___|**

District Sub-district Union

| **District** | **Sub-district** | **Union** |
| --- | --- | --- |
| Magura=1 | Shreepur=1 | Shreepur Sadar=1 |
|  |  | Shreekhol=2 |
|  | Magura Sadar=2 | Chaulia=1 |
|  |  | Bogiya=2 |
| Jessore=2 | Jhikargacha=1 | Jhikorgacha Sadar=1 |
|  |  | Godkhali=2 |
|  | Sharsha=2 | Sharsha Sadar=1 |
|  |  | Benapole=2 |
| Khulna=3 | Rupsa=1 | Ghatvog=1 |
|  |  | TSB=2 |
|  | Fultola=2 | Fultola Sadar=1 |
|  |  | Atragiletola=2 |

- **Please check One:**

| □ Shasthya Shebika  □ Shasthya Kormi  □ Program Organizer  □ Lab Program Organizer  □ Drug Seller | □ Village Doctor  □ DOTS Provider  □ Imam  □ Muezzin  □ Folk song and popular theatre group | □ UP Member  □ Cured TB Patient  □ Businessman`  □ Social worker  □ Factory Worker  □ Head Master  □ Others………………… |
| --- | --- | --- |

| Signature of the Enumerator with Date  ___________________  [ensure that all parts of the questionniare is filled up]  Start time: --------- / ---------  Hour  Minute  End time: ---------- / --------  Minute  Hour | Signature of the Field Survervisor  Code checked  Field Random checked | Signature of the Data Entry Operator  _____________  [checked]  Problems in entering data  Section Question |
| --- | --- | --- |

| **Section I: General and Demographic Information** | | | | | |
| --- | --- | --- | --- | --- | --- |
| Sex* | Age | Marital status  ** | Educational Qualification*** | Occupation**** | Monthly income |
|  |  |  |  |  |  |
|  |  |  |  |  |  |
|  |  |  |  |  |  |
|  |  |  |  |  |  |
|  |  |  |  |  |  |
|  |  |  |  |  |  |
|  |  |  |  |  |  |
|  |  |  |  |  |  |
|  |  |  |  |  |  |
|  |  |  |  |  |  |

*Sex: ****Occupation code:

1=Male 2=Female

1= Agriculture (own farm)

2= Fishing

3= Business

4= Day Labor

5= Rickshaw/Van puller

6= Government salaried staff

7= Private/NGO salaried staff

8= Doctor (Homeopath, Allopath)

9= Self employed (Advocate, others)

10= Driver (Bus, Truck, automobile etc.)

11= House owner/shop owner

12= Housewife

13= Retired

14= Student

15= Unemployed

16= Voluntary

17= others (please specify)

…………………………………..

**Marital Status:

1= Married 2= Unmarried 3=Widow/widower

4= Divorced 5= Separated

***Educational qualification:

1= Never attended at school 2= Primary (class 1-5)

3= Secondary (class 6-10) 4=SSC/Dakhil/Equivalent

5=HSC/Fazil/Equivalent 6=Graduate

7= Masters/MBBS/Engineering 8= Diploma/Polytechnic

9= others (Please specify)…………………………………….

| **Section II: Knowledge questions** | | | |
| --- | --- | --- | --- |
| No | Questions | Response | Remarks |
| **Part A: Knowledge on TB** | | | |
| 1. 1. | Have you ever heard of Tuberculosis or TB? | Yes…………………………………................1  No………………………………..…...............2 | If no, end the questionnaire here |
| 1. 2. | What are the signs and symptoms of TB according to you? *(check all that apply)*   \|  \| Cough…………………………..……………………………………...…1 \|  \| \| --- \| --- \| --- \| \|  \| Cough lasting for 3 weeks or longer…………………………..…………2 \|  \| \|  \| Chest pain…………………………..……………………………...….…3 \|  \| \|  \| Coughing up blood in the sputum…………………………..……………4 \|  \| \|  \| Weakness…………………………..………………………………...…..5 \|  \| \|  \| Fatigue…………………………..…………………………………….…6 \|  \| \|  \| Weight loss…………………………..…………………………..…...….7 \|  \| \|  \| Loss of appetite…………………………..…………………………...….8 \|  \| \|  \| Shortness of breath…………………………..…………………………...9 \|  \| \|  \| Chills…………………………..……………………………………..…10 \|  \| \|  \| Fever…………………………..……………………………………..…11 \|  \| \|  \| Sweating at night…………………………..………………………..….12 \|  \| \|  \| Others (Please specify)…………………………………………………… \|  \| | |  |
| 1. 3. | After how many days if above mentioned symptoms persist, one should seek treatment? | Immediate…………………………..…………1  More than 3 weeks…………………..………..2  More than 7 days…………………..………….3  No treatment necessary……………..………...4  Others (Please specify………………...………5 |  |
|  | How long a person should take treatment to get cured from TB? | Less than 6 months…………………………....1  6 months……………………………………....1  More than 6 months………………………..…6  Don’t know………………………..................99 |  |
|  | Do you think TB can be cured once treated? | Yes……………………………………..……...1  No…………………………………...………...2  Don’t know……………………………..……99 |  |
|  | Do you think early treatment is important once TB is suspected? | Yes……………………………………….……1  No……………………………..........................2  Don’t know……………………………….….99 |  |
| 1. 4. | How can TB be transmitted? | Contact with TB patients……………………...1  Air droplets…………………………………....2  Living in the same house within close proximity…………………………………..….3  Sharing utensils………………………….…....4  Malnutrition………………………….…….….5  Unhealthy environment…………………...…..6  Unawareness……………………………….....7  Others…………………………………………8  Please specify:  …………………………………………………. |  |
| 1. 5. | Do you think TB can be transmitted during treatment? | Yes…………………………………….………1  No………………………………….. ………...2  Don’t know…………………………….…….99 |  |
|  | Can a person have TB more than once in lifetime? | Yes…………………………………….………1  No…………………………………..................2  Don’t know…………………………………..99 |  |
| 1. 10. | Can children have TB too? | Yes………………………………………….…1  No………………………………….. ………...2  Don’t know……………………………….….99 |  |
| **Part B: Source of knowledge on TB** | | | |
| 1. | Where did you learn about TB? *(check all that apply)*   \|  \| Television………………………..…………………………………….…1 \|  \| \| --- \| --- \| --- \| \|  \| Radio …………………………..……………………………………...…2 \|  \| \|  \| Newspaper…………………………..………………………………...…3 \|  \| \|  \| Billboard/Poster…………………………..………………………...……4 \|  \| \|  \| Government health staff…………………………..…………………...…5 \|  \| \| 1. B \| BRAC health staff…………………………..………………………...…6 \|  \| \|  \| Other non-governmental organizations…………………………..…...…7 \|  \| \|  \| Private clinic…………………………..……………………………...….8 \|  \| \|  \| Community people…………………………..………………………...…9 \|  \| \|  \| Cured TB patient…………………………..………………………...….10 \|  \| \|  \| Others (Please specify)…………………………………………………… \|  \| | |  |
