# Supplementary material for: Knowledge and attitude of key community members towards tuberculosis: mixed method study from BRAC TB control areas in Bangladesh
Source: BMC Public Health. 2015 Jan 31;15:52. doi: 10.1186/s12889-015-1390-5 (PMC4322444; doi:10.1186/s12889-015-1390-5)
Supplement: Additional file 3: — Reported responses of key community members regarding knowledge on TB, doc, descriptive report on frequency of all correct and incorrect responses for each of the 10 questions used for assessing knowledge about TB. [file 12889_2015_1390_MOESM3_ESM.docx]

| **Table 1- Reported responses of key community members regarding knowledge on TB (n=432)** | | | | | |
| --- | --- | --- | --- | --- | --- |
| Question | Response | Category of the key community members n(%) | | | |
|  |  | BRAC worker | Informal healthcare provider | Community leader | Community member |
| Have you ever heard of TB? | Correct | 224(99) | 72(99) | 73(99) | 60(100) |
|  | Incorrect | 1(1) | 1(1) | 1(1) | 0(0) |
| What are the signs and symptoms of TB? | Correct | 205(91) | 57(78) | 46(62) | 46(77) |
|  | Incorrect | 20(9) | 16(22) | 28(38) | 14(23) |
| After how many days one should seek treatment for TB? | Correct | 194(86) | 64(88) | 61(82) | 51(85) |
|  | Incorrect | 31(14) | 9(12) | 13(18) | 9(15) |
| How long a person should take treatment to get cured from TB? | Correct | 207(92) | 64(88) | 63(85) | 50(83) |
|  | Incorrect | 18(8) | 9(12) | 11(15) | 10(17) |
| Do you think TB can be cured once treated? | Correct | 225(100) | 72(99) | 74(100) | 60(100) |
|  | Incorrect | 0(0) | 1(1) | 0(0) | 0(0) |
| Do you think early treatment is important once TB is suspected? | Correct | 225(100) | 72(99) | 74(100) | 60(100) |
|  | Incorrect | 0(0) | 1(1) | 0(0) | 0(0) |
| How can TB be transmitted? | Correct | 225(100) | 73(100) | 74(100) | 60(100) |
|  | Incorrect | 0(0) | 0(0) | 0(0) | 0(0) |
| Do you think TB can be transmitted during treatment? | Correct | 225(100) | 71(97) | 69(93) | 57(95) |
|  | Incorrect | 0(0) | 2(3) | 5(7) | 3(5) |
| Can a person have TB more than once in lifetime? | Correct | 206(92) | 67(92) | 56(76) | 44(73) |
|  | Incorrect | 19(8) | 6(8) | 18(24) | 16(27) |
| Can children have TB too? | Correct | 203(90) | 59(81) | 56(76) | 42(70) |
|  | Incorrect | 22(10) | 14(19) | 18(24) | 18(30) |
